# Insights into Body Size Evolution: A Comparative Transcriptome Study on Three Species of Asian Sisoridae Catfish

**DOI:** 10.3390/ijms20040944

**Published:** 2019-02-21

**Authors:** Wansheng Jiang, Yicheng Guo, Kunfeng Yang, Qiong Shi, Junxing Yang

**Affiliations:** 1 State Key Laboratory of Genetic Resources and Evolution, Kunming Institute of Zoology, Chinese Academy of Sciences, Kunming 650223, China; jiangws@mail.kiz.ac.cn (W.J.); guoyc2016@gmail.com (Y.G.); 18213848413@163.com (K.Y.); 2 Yunnan Key Laboratory of Plateau Fish Breeding, Kunming Institute of Zoology, Chinese Academy of Sciences, Kunming 650223, China; 3 Shenzhen Key Lab of Marine Genomics, Guangdong Provincial Key Lab of Molecular Breeding in Marine Economic Animals, BGI Academy of Marine Sciences, BGI Marine, BGI, Shenzhen 5180083, China

**Keywords:** *Bagarius*, body size, growth, ribosome, pyruvate metabolic

## Abstract

Body size is one of the most important attributes of a species, but the basic question of why and how each species reaches a different “right size” is still largely unknown. Herein, three phylogenetically closely related catfishes from Sisoridae, including one extraordinarily large-sized *Bagarius yarrelli* and two average-sized *Glyptothorax macromaculatus* and *Oreoglanis setiger*, were comparatively studied using RNA-Seq. Approximately 17,000 protein-coding genes were annotated for each of the three fishes, and 9509 genes were identified as high-confidence orthologous gene pairs. Comparative expressions uncovered a similar functional cluster about ribosome biogenesis was enriched in different tissues of the upregulated genes of *Bagarius yarrelli*. Moreover, differentially expressed genes and positively selected genes revealed that the glycolysis/pyruvate metabolism and cell cycle pathways have also greatly enhanced in this large-sized species. In total, 20 size-related candidate genes (including two growth modulators: the serine/threonine-protein kinases 3 (*AKT3*) and adaptor protein 1 (*SH2B1*), and a crucial pyruvate kinase (*PKM2A*)) were identified by multiplying comparative analyses along with gene functional screening, which would play major roles in enabling the large body size associated with *Bagarius yarrelli* and provide new insights into body size evolution. In conjunction with field observations and morphological comparisons, we hypothesize that habitat preferences promote size divergence of sisorids.

## 1. Introduction

In 1928, Haldane stated that: “the most obvious differences between different animals are differences of size, but, for some reason, the zoologists have paid singularly little attention to them” [1]. After nearly a century later, this statement still holds true to some degree. Body size is perhaps the most important attribute of a species [2], with the largest metazoans (e.g., whales and giant sequoias) weighing over 21 orders of magnitude more than the smallest microbes [3]. Body size differences can be easily viewed across the biosphere; however, the mechanisms that determine body size, especially those selecting the “right size” for a specific species, are largely unknown [4,5]. The factors that determine body size remain a mystery [6].

Previous studies on size issues, in the context of evolutionary genetics, have usually been conducted based on model organisms such as worms, flies, mice, and humans. There is now evidence that differences in body size among species can essentially reflect differences in cell number, cell size, or both [5]. In the animal kingdom, the large differences in body size observed in vertebrates (such as between mice and humans) mainly result from variations in cell number [6]; whereas, in invertebrates, cell size significantly contributed to body size differences, such as between worms (*Caenorhabditis elegans*) [7]; and flies (*Drosophila* spp.) [8]. Cell number is usually determined through the balance between cell proliferation and cell death, and cell size generally depends on cell growth [5]. While age and environmental factors such as food intake may affect individual body size, both cell number and cell size are predominantly determined by genetic factors [4]. Several pathways have been shown to be associated with body size, and some of these are evolutionarily conserved or functionally similar from invertebrates to vertebrates, such as Hippo, the transforming growth factor β (*TGFβ*) superfamily, and the most comprehensive insulin/PI3-kinase (*PI3K*)/serine-threonine kinase (*AKT*)/target of rapamycin (*TOR*) signaling pathways [5,9].

The mystery of body size evolution among non-model species, however, is poorly understood. As a matter of fact, the overwhelming diversity in body size stems from interspecies variations, such as between mice and elephants; however, these are generally distantly related in phylogeny. When examining phylogenetically closely related groups, such as mice and other rodents, similar body sizes are noted. While considering the phylogenetic relationships in a relative scale, distantly related species are usually differently-sized and have diverse habits and habitats, but closely related species are often similarly-sized and share similar life strategies. For instance, a comparative analysis of the New World terrestrial Carnivora revealed a strong phylogenetic pattern for body size [10]. This general pattern of the interrelationship of phylogeny and body size, however, may be disrupted by evolutionary exceptions or extremes. Although infrequent, some phylogenetically closely related species have also evolved significantly divergent body sizes.

**Figure 1:**
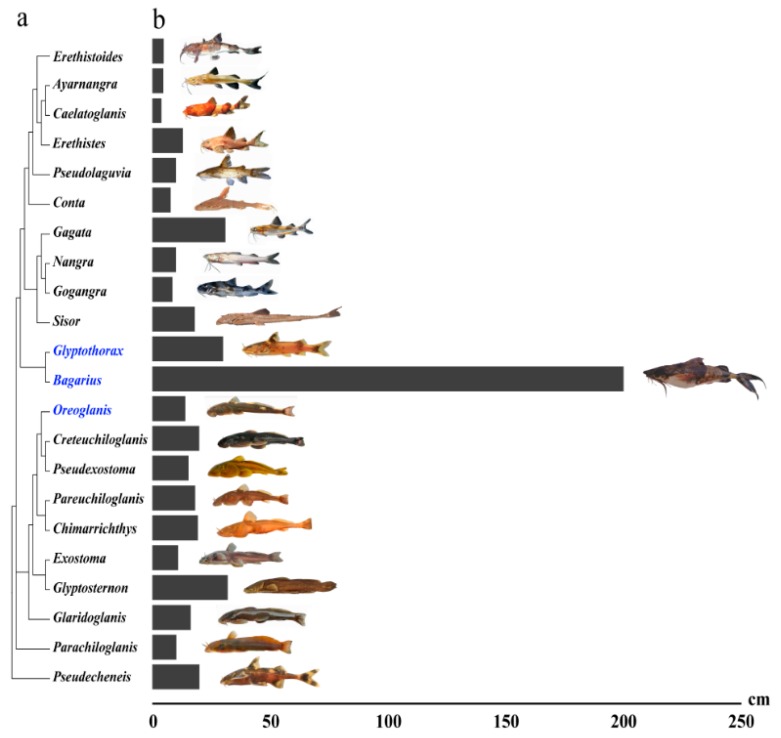
Intergeneric phylogenetic relationships and body length differences in Sisoridae. (**a**) phylogenetic relationships recreated based on a previous study [13]; (**b**) the maximum body length recorded for each genus based on Fishbase (http://www.fishbase.org/search.php). The photographs present a typical appearance for each genus, among which *Conta*, *Erethistes*, *Erethistoides*, *Gogangra*, *Nangra*, *Pseudolaguvia*, and *Sisor* were provided by courtesy of Heok Hee Ng, *Ayarnangra*, *Caelatoglanis*, and *Gagata* by courtesy of Kamphol Udomritthiruj, *Parachiloglanis* by courtesy of Ryan Thoni, and the others were taken by the first author (Wansheng Jiang). The genera that include the species in this study are highlighted in blue.

Sisoridae, which comprises the largest family of Asian catfish, is a group restrictively living in the southeastern Himalayas, with more than 200 species in 22 genera [11]. Sisoridae members are commonly seen in hill streams, with a general body size under 30 cm standard length (SL), with most around 10 cm. However, one exception does occur in the genus *Bagarius* (giant devil catfish), which usually lives in great rivers, and can reach extraordinary lengths of up to 2 m SL (Figure 1; [11]). Although the taxonomic and phylogenetic relationships of *Bagarius* remain unclear, the large body size of *Bagarius* seems to have been derived from the origin or early evolutionary stage of this genus and then fixated along speciation because large-sized species widely span its distribution areas, from the Indus drainage (in Pakistan and India), east to the Red River (China and Vietnam), and south to the Malay Peninsula and Indonesia [12]. The extraordinary body size of *Bagarius* also does not appear to be a terminus of a gradual phylogenetic increase, with the morphological and molecular intrafamilial phylogenetic relationships validating that *Glyptothorax*, its phylogenetic sister group, is a typically small-bodied genus (Figure 1; [11,13]). Hence, *Bagarius* species and their phylogenetically related confamilial species could be ideal to study the genetic mechanisms of body size evolution.

In this study, three non-model catfish species from three distinct genera in Sisoridae were collected: *Bagarius yarrelli* (BY), *Glyptothorax macromaculatus* (GM), and *Oreoglanis setiger* (OS). BY can grow to 2 m [11], which represents the extremely large size, whereas GM and OS are typically small-sized species, with a maximum SL of up to 20 cm. All three species are distributed in the Lancangjiang River (upstream of Mekong River), and thus could have evolved under a similar environmental background along river evolvement. BY and GM were selected because these are closely related sister genera, whereas OS represents a more distantly related genus (Figure 1). To investigate the genetic basis underlying body size evolution in these three species, we investigated gene expression in brain, liver, and muscle based on the fact that these major organs are involved in growth. The brain is a centralized organ for driving secretion of various hormones for growth, the liver is critical for its major roles in metabolism, and muscle is a major effector target of growth, as well as a secretory organ for crosstalk with other tissues [14,15].

To provide the first insights into body size evolution among Sisoridae, we performed RNA-Seq on the three species to identify the differentially expressed genes (DEGs) in the brain, liver and muscle tissues, and to explore fast-evolving genes (FEGs) and positively selected genes (PSGs) based on the putative one-to-one orthologs. We investigated tissue-specific transcriptomic patterns, and considering that global growth involves the coordination of cellular responses among different tissues, we focused on DEGs with similar profiles among various tissues. We hypothesized that the large-sized BY species would show some gene expression, sequence mutation, or both, which are associated with body size evolution when compared to the small-sized species GM and OS. We predicted that we would find some BY-specific enriched pathways and would screen some candidate genes that were implicated in cell growth, proliferation, or other relevant functions. To the best of our knowledge, this is the first comparative transcriptome study on body size evolution based on three closely related non-model fishes. This work may improve our understanding of body size evolution.

## 2. Results

### 2.1. Length Frequency and Preliminary Age Estimations of BY, GM, and OS

**Figure 2:**
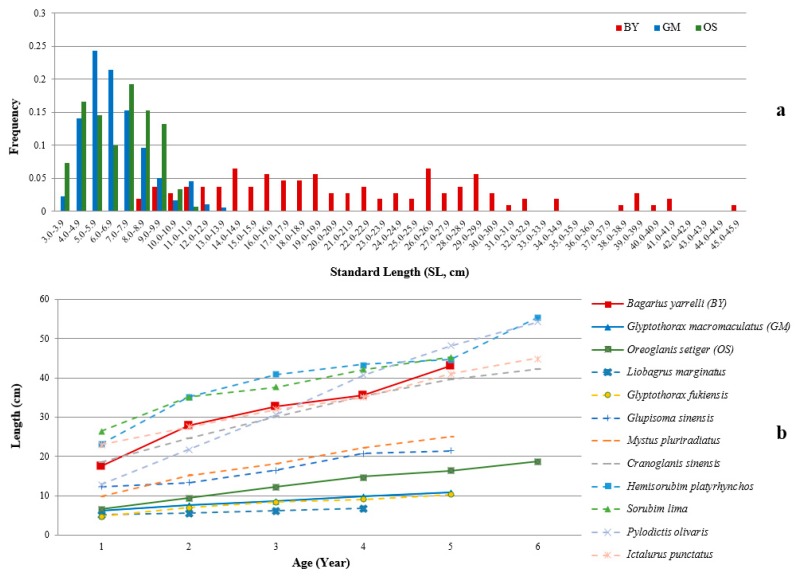
Length frequency distributions and preliminary age estimations of *Bagarius yarrelli* (BY), *Glyptothorax macromaculatus* (GM), and *Oreoglanis setiger* (OS). (**a**) length frequency of the specimens deposited in the Kunming Natural History Museum of Zoology, Chinese Academy of Sciences; (**b**) age–length relationship of BY, GM, OS (solid line) and other eight catfish species (dotted line).

According to the specimens collected over a span of 30 years in Kunming Natural History Museum of Zoology, Chinese Academy of Sciences (KNHM), the length frequency of BY, GM, and OS showed differences in patterns (Figure 2a). The maximum SL values of BY, GM, and OS were 45.0, 13.6, and 11.5 cm, respectively. The length range distributions of large-sized species (BY) and small-sized species (GM and OS) were different, where the length of BY was distributed randomly across 8–45 cm, whereas the most frequent lengths of GM and OS were within the range of 4–9 cm. A preliminary age estimation of BY, GM, and OS also indicated that their growth rates had diverged (Figure 2b). Apparently, the large-sized species BY has a significantly higher growth rate. A literature search of age-length correlations among nine other catfish species (data trimmed to younger than six years old) also indicated a positive correlation between body size and growth rate, where the large-sized species (e.g., *Hemisorubim platyrhynchos* [16], *Sorubim lima* [17], *Pylodictis olivaris* [18], and *Ictalurus punctatus* [19]), the medium-sized species (e.g., *Mystus pluriradiatus* [20], and *Glupisoma sinensis* [21]), and the small-sized species (e.g., *Liobagrus marginatus* [22], and *Glyptothorax fukiensis* [23]) had relatively fast, medium, and slow growth rates, respectively. Although we still have a lot of unknowns related to the growth and age of the BY, GM, and OS species, to guide an early-life sampling strategy, we only collected fish estimated under three years old, approximately with an SL < 32 cm in BY, and < 9 cm in GM and OS. According to the length frequency of these three species, we assumed that individuals less than three years old were continuously growing young fish, not non-growing mature fish.

### 2.2. External Morphology Comparison of BY, GM, and OS

**Figure 3:**
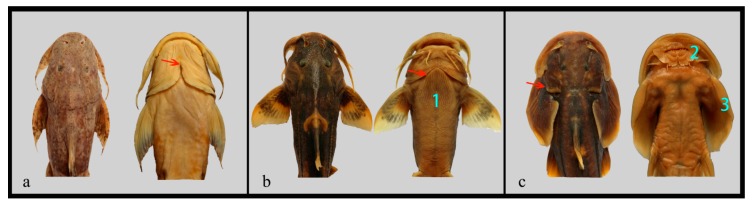
The front half body view of BY (**a**), GM (**b**), and OS (**c**). Arrows indicate the end of gill openings, and the numbers indicate the modified epidermal adhesive structures as follows: (1) thoracic adhesive apparatus; (2) adhesive sucker; and (3) adhesive unbranched rays.

An external morphological examination showed these three species differ distinctly in terms of gill openings and adhesive structures. The large-sized species BY has uniquely broad gill openings that disconnect the gill membrane from the isthmus (Figure 3a). For the small-sized species GM and OS, however, the gill openings are either much narrower (GM, Figure 3b) or completely degenerated at the ventral surface of head (OS, Figure 3c). The large-sized BY does not have any specialized structures with adhesive functions on the ventral side. In contrast, small-sized species have evolved highly modified epidermal structures such as the thoracic adhesive apparatus (GM, Figure 3b) or adhesive sucker (OS, Figure 3c) to adhere to the substrate of rheophilic habitats.

### 2.3. De Novo Assembly and Annotation of the Three Transcriptomes

To explore the genetic basis of large body size in BY between GM and OS, the brain, liver, and muscle tissues from each of the BY, GM, and OS samples (*n* = 3 per group) were examined by RNA-Seq analysis, from which we obtained 891,062,592 raw reads, with 802,660,430 clean reads generated after quality filtering to yield 202 gigabases (GB) of clean nucleotides (Table S1). These clean reads were then used for de novo transcriptome assembly. A total of 611,175 transcripts were obtained for BY; 533,812 for GM; and 615,344 for OS, with contig N50s for all of the samples generally ranging from 818 bp to 1135 bp (Table S2). Using Trinotate, a total of 64,091 (11%, BY), 62,539 (12%, GM), and 54,905 (9%, OS) transcripts were annotated with 14,927 (BY), 14,944 (GM), and 13,937 (OS) genes, respectively. After combining the results of homology searching to the protein databases of channel fish and zebrafish, we finally identified 18,935 (BY), 19,186 (GM), and 16,872 (OS) protein-coding genes, which covered over 65% of the known protein-coding genes of both channel fish and zebrafish (ca. 26,000, [24,25]). Among these genes, 10,436 for BY, 10,606 for GM, and 9248 for OS were mapped to zebrafish Gene Ontology (GO) categories (Tables S3–S5). A total of 51 terms were identified within the main GO categories and included cellular component (13), biological process (23), and molecular function (15; Figure S2) using the WEGO online web tool. However, no GO terms were significantly different among the three species.

### 2.4. DEGs in BY Relative to GM and OS

**Figure 4:**
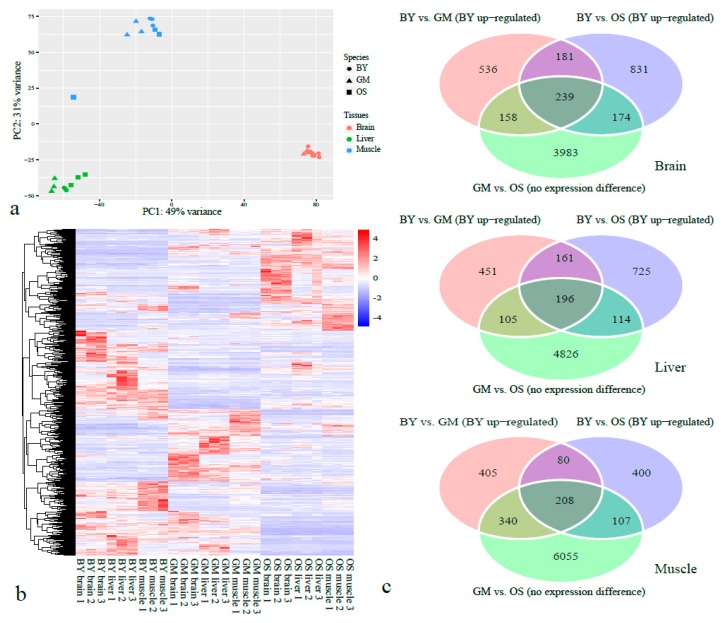
Overview of the expression differentially expressed genes (DEGs) identified in BY relative to GM and OS species. (**a**) the principal component analysis of the overall gene expression among three tissues in three species; (**b**) a heatmap of the overlapped DEGs between any of the two in six DEG datasets. The six DEG datasets were obtained from the comparisons between BY vs. GM and BY vs. OS in three tissues; (**c**) Venn diagrams of upregulated BY DEGs when compared with GM and OS samples for all three types of tissues, with the overlapping genes showing no statistical transcriptional differences between GM and OS.

The overall gene expression patterns of these three species were clustered by tissues (Figure 4a), which indicated that the tissue-specific expression genes were abundant in the overall expression data. A total of 5877 union sets of genes were identified as DEGs in BY in any of the three tissues, which were obtained from both the BY vs. GM comparison (3740 DEGs) and BY vs. OS comparison (4295 DEGs). Among the DEGs between BY vs. GM and BY vs. OS comparisons of three tissues (a total of six DEGs datasets), 1254 genes showed overlaps between any of the two DEG datasets. The gene expression heatmap of these 1254 overlapped DEGs showed that they clustered in different tissues by species (Figure 4b), which would suggest that these genes are related to the species-specific phenotypic differences. As we mainly focused on the BY-specific expression patterns, only the BY upregulated DEGs were emphasized in the subsequent analyses.

When considering the BY upregulated DEGs, a total of 1114 (brain), 913 (liver), and 1033 (muscle) were detected in the BY vs. GM comparison, and 1425 (brain), 1193 (liver), and 795 (muscle) were detected in the BY vs. OS comparison (Figure 4c). All of the above six BY upregulated DEGs lists were inputted independently to Database for Annotation, Visualization and Integrated Discovery (DAVID) for functional annotation analysis. Interestingly, one universally identical functional cluster was observed across all six independent BY upregulated DEGs lists. This pertained to ribosome biogenesis and included the terms ribosome (GO:0005840 and dre03010), structural constituent of ribosome (GO:0003735), intracellular ribonucleoprotein complex (GO:0030529), and translation (GO:0006412) (Table S6). A tissue-specific common cluster was generated from the liver samples of both the BY vs. GM (ES = 2.4) and BY vs. OS (ES = 3.1) comparisons, which was functionally related to cell proliferation and included the terms cell cycle (GO:0007049), cell division (GO:0051301), and mitotic nuclear division (GO:0007067) (Table S6).

To narrow down the BY upregulated DEGs, we only selected the overlapping genes between BY vs. GM and BY vs. OS comparisons, where 420, 357, and 288 genes were detected in the brain, liver, and muscle, respectively (Figure 4c). DAVID analysis based on these three reduced DEG lists grouped considerably fewer functional clusters; however, similar ribosome biogenesis-related clusters were still revealed from brain and muscle samples, including GO terms and pathways, such as ribosome (GO:0005840 and dre03010) and structural constituent of the ribosome (GO:0003735) (Table S7). In spite of a slightly lower score (ES = 1.2), a functional cluster pertaining to cell proliferation was also revealed from the liver-specific DEG list, including three identical GO terms (Table S7).

When we further narrowed down the BY upregulated overlapping DEGs between BY vs. GM and BY vs. OS comparisons, by retaining these without transcriptional difference between GM and OS, only 239, 196, and 208 were retained for the brain, liver, and muscle, respectively (Figure 4c). No other similar enriched functional clusters were revealed from these tissues. However, some enriched terms were associated with functional importance, such as the FoxO signaling pathway (dre04068) and glycolytic process (GO:0006096; Table S7). In the enriched GO term of glycolytic process, fructose–bisphosphate aldolase C–A (*ALDOCA*), phosphofructokinase, muscle a (*PFKMA*), and pyruvate kinase, liver and red cell (*PKLR*) were found to be involved (Table S7).

### 2.5. FEGs and PSGs Involved in BY

After completing annotations for the three catfish species examined, 9509 genes were retained as high-confidence orthologous gene pairs. The pairwise distance between BY and GM was 0.072, and that between BY and OS was 0.091. The neighbor-joining (NJ) tree based on these orthologs showed the same relationship of these three species in Figure 1.

Median dN/dS values for genes in each GO category were used to represent the dN/dS value of GO category, and a total of 15 GO categories had significantly higher dN/dS values in BY relative to GM (*p* < 0.05, binomial test), thus representing having FEGs (Table S8). These GO categories harboring FEGs related to cell growth and proliferation and included the regulation of mitotic cell cycle (GO:0007346), hypothalamus development (GO:0021854), the oxidation–reduction process (GO:0055114), structural constituent of ribosome (GO:0003735), insulin receptor binding (GO:0005158), and insulin receptor signaling pathway (GO:0008286; Figure 5a, Table S8).

Furthermore, 63 BY genes (0.66%) were found to be under positive selection (Table S9). DAVID analysis revealed that among the PSGs, DNA repair protein RAD51 homolog 3 (*RAD51C*) and meiotic nuclear division protein 1 homolog (*MND1*) were annotated in reciprocal meiotic recombination (GO:0007131); tumor necrosis factor receptor superfamily member 1A (*TNFRSF1A*), *TGFβ3*, and Bcl-2-like protein 10 (*BCL2L10*) were annotated in the regulation of the apoptotic process (GO:0042981); and Hexokinase-2 (*HK2*) and pyruvate kinase, PKM (*PKM2A*) were annotated in the glycolytic process (GO:0006096). Additionally, G2/mitotic-specific cyclin-B2 (*CCNB2*), *TGFβ3*, and growth arrest and DNA damage 45 beta (*GADD45BA*) were simultaneously annotated in the cell cycle (dre04110) and FoxO signaling (dre04068) pathways (Table S9).

### 2.6. Positive Selection of PKM2A in BY

**Figure 5:**
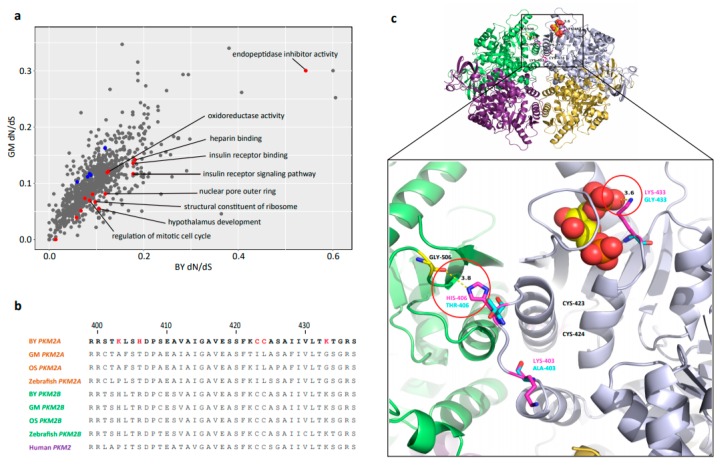
Fast-evolving genes (FEGs) enrichment and *PKM2A* sequence alignment and protein structure. (**a**) comparison of dN/dS ratios between BY and GM using gene ontology (GO) functional categories; highlighted red dots indicate GO terms with elevated evolutionary rates in BY that may be related to growth and proliferation, and blue dots indicate these GO terms with reduced evolutionary rates in BY; (**b**) sequence alignments of *PKM2A* & *PKM2B* between BY (red indicates positive selection sites), GM, OS, and zebrafish, with human *PKM2* included; (**c**) the construction of the *PKM2A* protein of BY, GM, and OS, with human *PKM2* used as a template (PDB id: 3srd). A possible hydrogen bond between H406 and G506, and an affinity enhancement between K433 and FBP (the red and yellow spheres) via the charge attraction of BY (in magenta) can be seen in the red cycle; no similar link was found between GM and OS (in cyan).

When utilizing the most rigorous BY upregulated DEGs list, the enriched GO term glycolytic process (GO:0006096; DAVID) was identified (Table S7). While using the PSG list for enrichment analysis, the same GO term glycolytic process (GO:0006096) was also identified, including the *HK2* and *PKM2A* genes (Table S9). Based on these findings, *PKM2A*, a crucial pyruvate kinase, was further examined. Upon examination of the protein structure, five positive selective sites were identified in BY relative to OS and GM (Figure 5b), with the BY *PKM2A* protein structure constructed using the human *PKM2* protein as the template (PDB id: 3srd). Interestingly, the first four residues (K403, H406, C423, and C424) were located within the C-C interface of the *PKM2A* tetramer, whereas the last one (K433) was located in the binding pocket of fructose-1,6-bisphosphate (FBP), a metabolic regulator [26]. According to this structure, the H406 residue of BY could form a hydrogen bond with a G506 residue located on another *PKM2A* monomer (Figure 5c; distance = 3.8 Å). The K433 residue is positively charged in vivo and is positioned near a negatively charged phosphate group, such as FBP (Figure 5c).

### 2.7. Screening for Genes of Interest and Candidate Genes

When further analyzing the BY upregulated DEGs that overlapped between BY vs. GM and BY vs. OS comparisons, we found 187 upregulated DEGs were identified in at least two of the brain, liver, and muscle tissues, while 56 DEGs were consistently co-upregulated in all three tissue samples. These co-upregulated BY DEGs across multiple tissues would reflect the BY-specific genetic basis at a global level. In addition to the BY PSGs, we treated them as the most interesting gene dataset worthy of exploring (Table S10). This gene list was further examined using the UniProt Knowledgebase [27] to search for every proven function that may relate to cell growth, proliferation, or other size-relevant importance. These genes were considered to have possible associations with the extreme BY body size and were narrowed down to 16 upregulated DEGs and four PSGs serving as candidate genes (Table 1). However, some important genes that were transcribed in just a single tissue would be theoretically excluded from the candidate genes from this screening strategy, such as the liver-specific pyruvate kinase *PKLR* and muscle-specific Phosphofructokinase *PFKMA*; these genes can only be concerned in the subsequent section in Discussion.

Among all these candidate genes, for instance, *AKT3* and adaptor protein 1 (*SH2B1*) are two important growth related modulators that can regulate growth, proliferation, and biosynthetic processes at multiple levels; ribosomal protein S3 (*RPS3*) is a multi-functional ribosomal protein; heat shock cognate 70-kd protein and tandem duplicate 2 (*HSP70.2*) and its carrier protein (*HIKESHI*) can regulate many processes including metabolism, cell proliferation and growth; polycomb group ring finger 1 *(PCGF1)*, transcription factor Dp-2 *(TFDP2*), *TGFβ3*, *CCNB2* are all functionally relevant to the cell cycle; and *PKM2A* and *HK2* are two important kinases in the process of glycolysis (see more details in the Discussion).

### 2.8. qRT-PCR Validation of Candidate Genes

To assess the overall reliability of the transcriptome data and RNA-Seq derived DEGs, half of the 20 candidate genes (*AKT3*, *RPS3*, *HSP70.2*, *HIKESHI*, *PCGF1*, *NRF1*, *TRAF4*, *USP9X*, *TFB2M*, and *TFDP2*) were selected for qRT-PCR validation. Primer sequences are listed in Table S11. The qRT-PCR results correlated with the RNA-Seq results when comparing BY to GM or OS for the three different tissues (brain, liver, and muscle), with all of the correlation coefficients being higher than 0.8 (Table S12 and figures imbedded). These findings substantiated the RNA-Seq derived DEGs and showed that accurate transcriptome profiles were generated.

## 3. Discussion

Sisoridae, which is the largest Asian catfish family, has high phenotypic diversity, with variations in noted adhesive structures, body shapes, color patterns, and, most striking, body sizes (Figure 1). This diversity can serve as a useful resource for studying the relationship between phenotype and genotype during evolution. A major goal of evolutionary genetics is to provide a mechanistic account of the genetic basis of interspecific phenotypic variation, with gene expression patterns often playing key roles in the process of morphological evolution [28]. RNA-Seq has been widely employed as an effective tool in DEG discovery and has furthered our understanding of the genetic basis of many fundamental evolutionary questions. Furthermore, this approach can serve as an effective method when identifying coding mutations, such as in PSGs, especially in non-model species [29]. In this study, three species, including the extraordinarily large body size (BY) and generally small size (GM & OS), of Sisoridae catfish had their transcriptomes sequenced and de novo assembled in an effort to examine body size evolution. Each of the obtained transcriptome profiles contained approximately 600,000 transcripts, with a contig N50 of about 1000 bp. Approximately 60,000 (ca. 10%) of all these assembled transcripts could be annotated to about 17,000 protein-coding genes for the three studied species. Most other assembled transcripts (ca. 90%) failed to match known proteins, which could represent either false or low quality transcripts or potentially novel genes. The low percentage of assembled transcripts for annotations has also been revealed from other fishes, such as cichlid fishes *Amphilophus astorquii* (13%), *A. zaliosus* (12%, [30]), and larger yellow croaker (*Pseudosciaena crocea*, 20%, [31]). However, the absolute number of identified protein-coding genes of BY, GM, and OS (ca., 17,000) in this study has covered more than 65% of the known protein-coding genes of both channel catfish and zebrafish (ca. 26,000, [24,25]), which was similar as most other fishes with de novo transcriptome analyses, such as larger yellow croaker (15,192, [31]), European eel (*Anguilla Anguilla*, 16,200, [32]), and mud loach (*Misgurnus anguillicaudatus*, 17,336, [33]). Furthermore, the lengths of the generated sequences allowed for the high-confidence identification of orthologous genes, which can be useful in future evolutionary studies and applications.

Body size is a complicated characteristic that can be either oligogenic or polygenic, with nutrition-dependent or independent pathways also being factors. In *Caenorhabditis elegans*, gene mutations in three or more different pathways (*TGFβ*, spectrin, and calcineurin pathways) can determine body length [34]. However, in domestic dogs, size variations seem to be attributed to only the single-nucleotide polymorphism (SNP) haplotype of the insulin-like growth factor I (*IGF1*) gene [35]. Prior to this study, the possible genetic basis associated with the unusual body size variations among Sisoridae catfish (Figure 1) was completely unknown. To provide the first insights into body size evolution of these sisorids, a comparative transcriptome analysis based on RNA-Seq of BY, GM, and OS was employed to screen for any possible clues. The following clusters/pathways and candidate genes were highly implicated with the extraordinary enlarged size of BY, when compared with the other two average, small-sized species GM and OS.

### 3.1. Multistep Functional Clusters Converging to Ribosome Biogenesis

When examining the BY upregulated DEGs relative to GM and OS, the functional clusters from different DEGs lists revealed an extremely similar cluster converging to an association with ribosome biogenesis, with the about five enriched GO terms or pathways (Tables S6 and S7). Various upregulated ribosome proteins (*RP*) were involved in these terms and included both large and small ribosome subunit component proteins (*RPL* and *RPS*) and mitochondrial ribosomal subunit proteins (*RPM*) (Tables S6 and S7). Ribosomes are molecular factories that carry out protein synthesis, provide the basis for protein production, and drive cell growth [36]. The upregulation of ribosome synthesis in response to favorable conditions can allow a cell to grow faster [37]. Ribosome biogenesis is the result of a series of coordinated steps that occur in the nucleolus, with the *RP* numbers substantially reflecting cellular activity, growth, and proliferation [36,38]. Additionally, beyond the predominant functions of translation, some *PR* genes also have many extra-ribosomal roles, e.g., one of the candidate genes, *RPS3*, can regulate DNA repair, apoptosis, and post-translational modifications to affect cell growth and proliferation [39]. Many studies, although predominantly in cancers, have indicated that ribosome quantities control the G1–S-phase transition, thus regulating cell cycle progression in a *P53*-dependent (*RP*-*MDM2*-*P53* stress response pathway) or independent manner [38]. We intentionally examined the expression of two *RP* related proteins (*P53* and *MDM2*) among these three species. With the exception of an upregulation of *P53* in BY liver samples (log_2_ FC = 2.78, FDR = 0.002) when compared with OS, no other significant alterations in expression were observed. This may suggest that the *MDM2*-*P53* pathway was ‘off’ in BY, as common as other normal growing species [40], thus further indicating that BY growth does not occur in a tumor-like fashion. Whether the downstream response to the *RP* upregulation occurs in a *P53*-independent manner or if other extra-ribosomal functions regulate cell growth remains elusive. However, the common enrichment of a ribosome biogenesis-related cluster suggests that this could be a crucial module relevant to the large BY body size, as it would maintain higher levels of cell growth and proliferation.

### 3.2. Cell Proliferation Cluster and Metabolism Pathways

In addition to the common pattern of ribosome biogenesis, another identical cluster related to cell proliferation was revealed and it included the same three significantly enriched GO terms: cell cycle (GO:0007049), cell division (GO:0051301), and mitotic nuclear division (GO:0007067). Interestingly, this enriched functional cluster showed a liver-specific manner (Tables S6 and S7). Although we still have a lot to find out about cell signaling across tissues during proliferation, the cell cycle process of large-sized BY would possibly be enhanced, at least in the liver. Cell cycle and cell division, needless to say, are essential to cellular proliferation. Furthermore, BY FEGs were also enriched in the regulation of the mitotic cell cycle (GO:0007346; Figure 5a and Table S8), and two candidate PSGs, *CCNB2* and *TGFβ3*, were annotated in the cell cycle pathway (dre04110; Table S9). *CCNB2* is one of the two members of the B-type cyclin family (B1 and B2). *CCNB2* knockout mice, which develop normally, are smaller than normal mice, which suggests that *CCNB2* gives some growth advantage [41]. *TGFβ3* belongs to the well-known *TGFβ* superfamily that can regulate cellular processes of mice by increasing cyclin D1 (a marker of cell proliferation) expression [42]. *CCNB2* and *TGFβ3* under positive selection in this study warrant further examination.

Other significantly enriched GO terms, which may have been excluded from the functional clusters analyses, were identified in biological process terms related to substance and energy metabolism, such as amino acid, glycolytic, and fatty acid metabolism, and the mitochondrion, and GTPase activator activity (Tables S6 and S7). The substance and energy metabolism area associated with these identified components is indispensable in cell growth and proliferation as it provides the required materials and energies.

### 3.3. Glycolysis/Pyruvate Metabolic Process

Another interesting enriched functional group, which was revealed several times from BY upregulated DEGs when compared with GM and OS in different tissues, was assessed in terms of the function of the glycolytic process (GO:0006096) and pyruvate metabolism (dre00620) (Tables S6 and S7). These involved genes include some key kinases in the glycolysis/pyruvate metabolic process such as *PFKMA* and *PKLR* (DEGs), and *HK* and *PKM2A* (PSGs). Glycolysis is the fundamental metabolic pathway that contains a series of reactions that can extract energy from glucose to pyruvate, and then pyruvate acts as a key intersection in the network of the following substances and energy cycles. As the end product of glycolysis, pyruvate is usually transported into the mitochondria where it serves as a master fuel input for the tricarboxylic acid (TCA) cycle, thereby enabling ATP generation, and it drives several major biosynthetic pathways, including fatty acid, amino acid, and ATP metabolic processes [43].

Pyruvate kinase regulates the final rate-limiting step of glycolysis, which catalyzes the transfer of a phosphate group from phosphoenolpyruvate (PEP) to adenosine diphosphate (ADP), thereby yielding one molecule of pyruvate and one molecule of ATP [44]. The *PKLR* gene encodes both *PKL* and *PKR* transcripts by utilizing alternative promoters and differential splicing, while the *PKM* gene encodes *PKM1* and *PKM2*, which only differ by a single alternatively spliced exon [43]. In this study, the *PKLR* gene was found to be expressed specifically in the liver as expected, with the relative expression in BY being over eight times higher than those in both GM and OS (log_2_ FC > 3, *p* < 0.05).

*PKM2A*, as one of the two *PKM* genes in fish (the other one is *PKM2B*), was found to be under positive selection in BY. *PKM2* is the only pyruvate kinase that occurs in both a tetrameric and a dimeric form, with a high and low PEP (substrate) affinity, respectively [45]. The conformational changes of *PKM2* can regulate the rate of glycolysis, with the highly active tetrameric form favoring the degradation of glucose to CO_2_ and water via the TCA cycle or to lactate with the regeneration of energy [45]. According to the predictable protein structure of *PKM2* of BY (Figure 5c), the H406 residue could form a hydrogen bond with G506, thus enhancing the monomer association ability and enabling the formation of a more stable homo-tetramer. The K433 mutation could likely enhance the FBP binding affinity via charge attraction, thereby maintaining a highly active tetrameric form, which could substantially accelerate the catalytic efficiency of PEP [26]. Furthermore, *PKM2* is the only pyruvate kinase able to translocate to the nucleus where it conducts unique nuclear functions, such as acting as a protein-kinase-phosphorylating histone for gene transcription that directly controls cell-cycle progression and cell proliferation in human cells [46]. In short, the enhancement of pyruvate metabolism in BY via *PKLR* upregulation and the natural selection of *PKM2A* may strongly impact the regulation of other downstream targets and energy metabolic processes associated with body size.

### 3.4. Other Candidate Genes That May Influence the Large Body Size in BY

In addition to the BY-specific clusters, pathways, and related candidate genes mentioned above, some other size-relevant candidate genes that we revealed (Table 1) were also emphasized here. The first one is *AKT3*, which is one of the three isoforms (*AKT1*, *2*, and *3*) of the *AKT* family of serine/threonine protein kinases, also known as protein kinase B (*PKB*). *AKT* has been shown to be activated by a number of growth factors or cytokines in a *PI3K*-dependent manner, and it can mediate a broad variety of cellular responses, including cellular growth, proliferation, survival, and glucose metabolism [47]. *AKT3*, which is much less characterized than the two other *AKT* isoforms, has been shown to be functionally related to the cell growth of mice both in vitro [48] and in vivo [47]. Thus, it would seem that in relation to the large BY body size, *AKT3* may interact with the highly conserved growth-related *PI3K/AKT/TOR* pathway [5]. *AKT* is also a critical regulator in *PI3K/AKT* signaling and plays multiple roles in cell cycle progression [49], which is consistent with the DEG, FEG, and PSG findings (Tables S6–S9). Additionally, the upregulation of *AKT3* could modulate growth by regulating ribosome biogenesis at multiple levels to promote ribosomal RNA (rRNA) expression in human cells [50], which is reflected herein via the global enrichment of the cluster relating to ribosome biogenesis (Tables S6 and S7). Furthermore, *AKT3* can also impact glucose/pyruvate metabolism by increasing glucose uptake through glucose transporter type 1 (*GLUT1*), *HK*, or phosphofructo-1-kinase isozyme A (*PFK*) [51]. In the present study, glucose/pyruvate metabolism was enhanced in BY, with *PFKMA* and *PKLR* upregulated, and *HK2* and *PKM2A* under positive selection (Tables S6, S7 and S9, Figure 5b,c). In summary, *AKT3* may act as a key gene able to crosstalk with multiple downstream pathways to ultimately coordinate cellular growth, proliferation, and other biosynthetic processes in BY.

The second growth-related candidate gene of interest is *SH2B1*, which is one of three members of the *SH2B* family (*SH2B1*, *2*, and *3*), and it has a conserved structure with characteristic pleckstrin homology (PH) and Src homology 2 (SH2) domains [52]. The *SH2B* family serves as an adaptor protein that can mediate cell signaling in response to a variety of hormones, cytokines, and growth factors, including Janus kinase 2 (*JAK2*), growth hormone receptors (*GHR*), insulin receptor substrate-1, 2 (*IRS1*, *IRS2*), insulin receptors (*IR*), and insulin-like growth factor-1 receptors (*IGFR*), that can then positively mediate leptin and growth hormone (*GH*) (e.g., *JAK2*, *IRS1*, and/or *IRS2*) or insulin and *IGF-1* (e.g., *IRS1*, *IRS2*, *IR*, and *IGF1R*) signaling accordingly [52,53]. In *SH2B1*-stimulated *leptin*, *insulin*, and *IGF-1* signaling, the downstream *PI3K/AKT/TOR* signaling pathway can be activated by efficiently phosphorylating and prolonging the ability of the *IRS* protein [54]. While more remains to be uncovered regarding *SH2B1*, in mice, it has been shown to act as a key adaptor to several upstream activators of downstream effectors, and it is absolutely required for the maintenance of normal energy balance, body weight, and nutrient metabolism [52]. These findings suggest that, during the upregulation of BY *SH2B1*, it may serve as an adaptor function by binding to a broad range of key regulators that modulate the downstream signaling necessary for shaping an extraordinarily large size.

Other candidate genes that were not revealed directly from the DEG enrichment analyses, or that are not involved in a straightforward manner in the growth signals like *AKT3* and *SH2B1*, which may also play important roles in coordinating growth and corresponding metabolic processes, contribute especially to the global genetic basis of the body size evolution (Table 1). Among these is *HSP70.2*, which is one of the most important heat-shock protein (*HSP*) family members and plays an essential role in maintaining protein homeostasis in response heat shock, as well as to a large variety of environmental stresses [55]. *HIKESHI* is a nuclear import carrier of *HSP70* that can recognize *HSP70* in the cytoplasm, carry it into the nucleus, and then release it in the nucleus where it functions in protein protection [56]. Although more ecological studies need to be conducted, the consistent upregulation of both *HSP70* and its carrier, *HIKESHI*, seems to be relevant. In short, the 20 identified candidate genes (Table 1) were all functionally associated with cell growth, proliferation, or other necessary metabolic processes, which could further elucidate the mystery of body size evolution in BY.

### 3.5. Habitat Preferences May be Implicated in Body Size Evolution of BY

The extraordinarily enlarged body size of *Bagarius* could possibly provide a fitness advantage, however, ecological studies on this topic have been limited for a long time. We observed an interesting habitat preference trend among sisorids in field work: *Bagarius* species were usually found in the mainstreams of major rivers, but other small-sized sisorids were often sampled from hill-streamed tributaries. External morphological comparisons of BY, GM, and OS may be implicated in this observation (Figure 3). Most small-sized sisorids have evolved highly modified epidermal structures such as adhesive suckers or thoracic adhesive apparatus for adhering to the substrate in rheophilic tributaries [11]; correspondingly, their gill openings are narrow or completely degenerated on the ventral surface of the head (e.g., GM and OS, Figure 3b,c); however, *Bagarius* do not have any specialized adhesive functions on the ventral side, in contrast, they have uniquely broad gill openings that disconnect the gill membrane from the isthmus (e.g., BY, Figure 3a) [57]. A broader gill opening of *Bagarius* could diffuse water through the gill quickly, and maximize the intake of oxygen when cruising or hunting in mainstream rivers.

The habitats between the tributary and mainstream are generally different in terms of physical conditions such as width, depth, velocity, slope, sediment load, size of sediment debris, hydraulic roughness, and discharge [58]. These differences in physical conditions can also transfer to biotic conditions, which are easily reflected in the richness of macroinvertebrates and diversity of fish-rearing habitats [59]. Although not a strict comparison, the fish compositions of the tributaries and mainstream in the upper Mekong River, one of the main distribution areas of sisorids, are generally different [60]. Therefore, we propose an open hypothesis that habitat preferences, in combination with physiological differentiation and adaptation, would be involved in the large body size evolution of *Bagarius*.

The enhancement of the glycolysis/pyruvate metabolic process in BY found in this study might be crucial in promoting the physiological adaptation under the mainstream preferences of *Bagarius*. Food intake, naturally, would be one of the relevant but not investigated induction factors because dietary compositions are predictably different between mainstreams and tributaries [59,60]. Interestingly, catfish have usually been considered as carnivores; i.e., they should obtain their unique glucose needs mainly from animal diets with high protein and fat, but little carbohydrate contents through a process termed glycogenesis [61]. As a consequence, the efficiency of using limited glucose through the enhanced glycolytic/pyruvate metabolic process of BY would play an essential role in improving the overall energy flow. Further study could pay more attention to this aspect; however, the ecological or physiological studies about the unusual body size of *Bagarius* are all unexplored areas that await more investigation.

## 4. Materials and Methods

### 4.1. Laboratory Preparation

As only wild samples of BY, GM, and OS were available, a laboratorial preparation was performed before field collection. Considering that transcriptome profiles might vary with development stage among species, we employed an early-life sampling strategy for the three fish species to minimize possible age-induced variations. Because no age and body size correlations in these fishes were available, two independent methods were conducted to guide the early-life sampling strategy. To determine SL distribution frequency, we measured the SL of all the specimens of BY (*n* = 107), GM (*n* = 177), and OS (*n* = 151) that were deposited in the KNHM. All the specimens in KNHM were randomly collected over a span of 30 years, and we assumed that the SL distribution frequency of these specimens could largely represent that of natural populations. We also conducted preliminary age estimations of the three fish species. The left pectoral spines of BY (*n* = 10) and GM (*n* = 16) were disarticulated and cut into thin sections with a jeweler’s saw, embedded in epoxy resin, polished on very fine wet-dry sandpaper, and then examined under a binocular microscope to read the annuli. Because OS has no pectoral spine, we estimated its age by roughly referring to another glyptosternoid fish of similar body size [62]. Additionally, we conducted a literature search of other studies involving age estimation based on length in other catfish species. Furthermore, we also checked the morphological characters of these three species to identify other distinct traits associated with body size.

### 4.2. Fish and Tissue Sampling

BY, GM, and OS catfish were collected from the lower reaches of the Lancangjiang River (upper Mekong River) in Yunnan Province of China within a one-week period in June 2015 to minimize seasonal variations. An early-life sampling strategy was employed to reduce age variations based on the guidance of laboratory preparation, with only individuals estimated under three years of age included in this study (*n* = 3 per species). BY, GM, and OS were collected from the mainstream of the Lancangjiang River in Jinghong Prefecture, a left secondary tributary at Puer Prefecture, and a right tributary at Lincang Prefecture, respectively (Figure S1). All fish were captured by local fishermen using a backpack electro-shocker and then kept in a temporary fish tank for desensitizing overnight to alleviate the possible stress reaction caused by electrofishing. The fish were euthanized using MS-222 the next day, and then the brain, liver, and muscle tissues were collected and immediately nap frozen in liquid nitrogen. All samples were transferred and stored at −80 °C in the laboratory until analysis. All specimens were deposited in KNHM, and all animal experiments were approved by the Ethics and Experimental Animal Committee of the Kunming Institute of Zoology, Chinese Academy of Sciences (Approval ID: SMKX-2016024; approved on 15 December 2016).

### 4.3. RNA Extraction and Sequencing

Total RNA was extracted from each sample using TRlzol reagent (Invitrogen, Carlsbad, CA, USA) in conjunction with RNase-free DNaseI (TaKaRa, Shiga, Japan) according to the manufacturer’s protocols. RNA quality was examined using agarose gel electrophoresis and NanoDrop spectrophotometry (ThermoFisher Scientific, Waltham, MA, USA). RNA integrity and quantification were measured using an Agilent 2100 Bioanalyzer (Agilent, Santa Clara, CA, USA). Poly(A)-containing mRNAs were purified using oligo(dT)-attached magnetic beads and transcriptome libraries were generated using an Illumina TruSeq^TM^ RNA Sample Preparation kit (Illumina, San Diego, CA, USA) according to the manufacturer’s instructions. A cDNA library was constructed and purified using a QiaQuick PCR extraction kit (Qiagen, Valencia, CA, USA) and then enriched via PCR amplification. The complete libraries were sequenced using the Illumina HiSeq2500 system (Illumina, San Diego, CA, USA).

### 4.4. Transcriptome Assembly and Annotation

High-quality clean reads were obtained by removing the adaptor sequences, duplicated sequences, ambiguous reads (‘N’), and low-quality reads. A de novo reference transcriptome was constructed for each species by pooling conspecific libraries, and assembly was performed using Trinity (version 2.1.1, [63]) with default settings. Unigenes from Trinity assembly of the three species were then annotated using the Trinotate transcriptome annotation pipeline, with transcriptome coding regions predicted using the TransDecoder program (version 2.0.1, [64]). The Trinotate program utilized the UniProtKB/Swiss-Prot protein database as the reference protein database for Blastx and Blastp searching. The coding sequences of BY, GM and OS were predicted based on the assumption that the longest open reading frame in the longest transcript per gene had a greatest chance of being a protein-coding region. The longest transcripts were thus chosen to represent the genes for doing all the subsequent analyses.

Furthermore, protein-coding genes from channel catfish (*Ictalurus punctatus*, version IpCoco_1.2) and zebrafish (*Danio rerio*, version GRCz9) were also utilized for transcriptome annotations. Briefly, BLAST+ (version 2.6.0, https://blast.ncbi.nlm.nih.gov/Blast.cgi) was used to find reciprocal best hits among BY, OS, and GM with channel catfish and zebrafish, with the results then combined in Trinotate to determine the final annotation for each transcript. Genes were tentatively identified based on the best hits against known sequences. Assembled consensus sequences were used for Gene Ontology (GO; http://www.geneontology.org/) analyses. Web Gene Ontology Annotation Plot software (WEGO, http://wego.genomics.org.cn/) was employed to visualize, compare, and plot GO annotation results.

### 4.5. Identification of DEGs

For each individual library, the Illumina reads were mapped back to the appropriate transcriptome using Bowtie2 (version 2.2.6, [65]) within the RSEM program (version 1.2.25, [66]) to estimate the number of reads mapped to each contig. Transcription level normalization was performed using the expected count for each gene. Normalization was performed for all samples together using the trimmed mean of M-values method (TMM; function calcNormFactors in edgeR). Only genes with average expected count > 5 were included in the subsequent DEG analysis. Pairwise differential gene expression analyses for the same tissue were performed using the exactTest implemented in the edgeR package (version 3.16.5, [67]). This software was specifically developed and optimized to deal with over-dispersed count data produced by RNA-Seq. The ratio of the expected count between two samples was set as the fold change, and the FDR was obtained at the same time. Only orthologous genes with fold changes > 2 (log_2_ FC > 1) and FDR < 0.05 were defined as DEGs.

### 4.6. GO Annotations and Pathway Enrichment Analysis

Based on the identified DEGs, GO enrichment and Kyoto Encyclopedia of Genes and Genomes (KEGG) pathway analyses were performed using the functional annotation tool within the DAVID (version 6.8, [68]). In DAVID, the default setting of medium stringency was chosen to minimize the exclusion of potentially interesting terms. The terms (GO terms or KEGG pathways) with a modified Fisher exact *p*-value (EASE score) < 0.05 were considered to be significantly enriched terms of the DEG lists, and the clusters with enrichment scores > 1.3 (corresponding to *p* < 0.05) represented significantly enriched functional clusters of these enriched terms. The background gene list or identifiers were obtained from zebrafish, since they are widely used model organisms with a completely sequenced genome (http://zfin.org/).

### 4.7. Identification of FEGs and PSGs

Putative one-to-one orthologs were identified across the three catfish species through reciprocal best BLAST hit analysis. Gblocks [69] (version_0.91b; Parameters: −*t*  =  *c*, −*b3* =  1, −*b4*  =  6, and −*b5*  =  *n*) was utilized to reduce the rate of false positive predictions due to potential sequencing errors, incorrect alignments and non-orthologous regions based on codons. All of the one-to-one orthologous coding sequence (CDS) alignments were then combined into a super alignment and a NJ tree was constructed using MEGA7 [70]. Subsequently, this NJ tree was employed as the guiding tree to further identify FEGs and PSGs in PAML4 [71]. For estimating the evolutionary rate along a lineage, the values of nonsynonymous (dN), synonymous substitutions (dS), and the dN/dS ratio were calculated using the free-ratio model (model = 1) in the codeml program within PAML4. GO term data for FEGs analysis were downloaded from BioMart (http://www.biomart.org/), where GO categories with more than five orthologs were included in our analyses. Median values for orthologs were utilized as the evolutionary rates in each GO category. A binomial test was employed to identify different evolutionary rates between BY and GM. PSGs were detected using the improved branch-site likelihood method (model = 2) in PAML4, with the likelihood ratio test (LRT) test comparing the alternative model of positive selection on the background branch to a null model with no positive selection on the foreground branch for each orthologous gene. PSGs were inferred and obtained only when *p*-values < 0.05. Homology protein structure modeling of select PSG was performed using MODELLER (version 9.19, [72]), with a human orthologous protein used as the template.

### 4.8. Quantitative Real-Time PCR (qRT-PCR)

qRT-PCR was performed on 10 RNA-Seq derived DEGs (more details are provided in the Results). Primers were designed using Primer Premier 6 software (Premier Biosoft International, Palo Alto, CA, USA) to span an intro-exon boundary and generate an amplicon 100–200 bp in length, with sequences designed based on the consensus sequence of each alignment. The general cycling parameters employed were as follows: an initial denaturation at 95 °C for 5 min, followed by 45 cycles of denaturation at 95 °C for 10 s, annealing at 57 °C for 10 s, and extension at 72 °C for 10 s. All of the experiments were performed in triplicate to provide technical repeats. Relative expression levels were displayed as fold changes relative to an internal control gene, Eukaryotic translation elongation factor 2a (*EEF2A*), and they were calculated using the 2^−ΔΔ*C*T^ method [73].

### 4.9. Overview of Comparative Transcriptome Analyses

**Figure 6:**
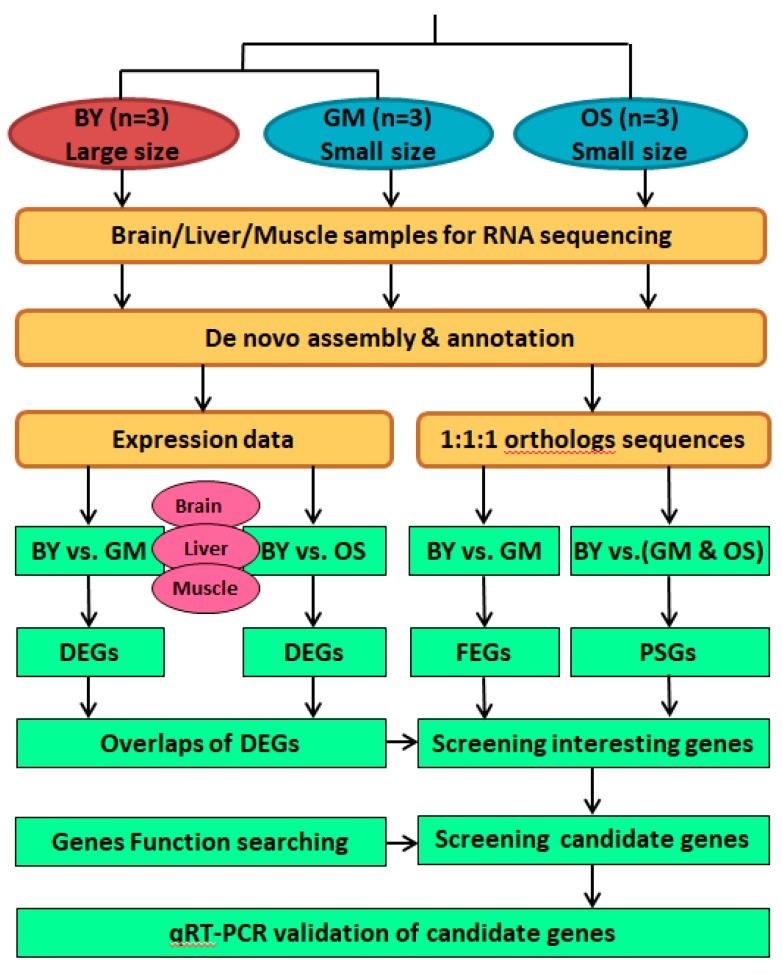
Flowchart that illustrating the comparative transcriptome analyses using RNA-Seq.

To organize the above methods in this study, a flowchart illustrating the overview of comparative transcriptome analyses is provided in Figure 6. In summary, the three reference transcriptomes of large-sized BY and small-sized GM and OS were assembled independently by pooling the conspecific libraries. For each species, the expression data of each library (three tissues in three individuals) were counted, normalized, and then employed for identifying the DEGs. The putative one-to-one orthologs were identified across the three species, and were then used in the identification of FEGs and PSGs. To obtain the BY-specific expression patterns in various tissues, progressive screening of DEGs was performed. Initially, we identified the BY upregulated DEGs by comparing BY vs. GM and BY vs. OS separately, with the goal of detecting similar DEG enrichment patterns. Additionally, we narrowed down the DEG lists by finding overlapped genes in BY vs. GM and BY vs. OS. These BY-specific overlapping DEGs may be attributed to their unique phenotypic feature of having an extraordinarily large size among Sisoridae. Furthermore, we determined the proportion of overlapping DEGs by searching for those with no transcriptional differences between GM and OS, which acted as the most rigorous condition to identify BY-specific expression patterns. Because evolutionary rates and the selection signals of coding genes might also contribute to the large BY body size, FEGs and PSGs were examined by counting the BY-specific mutation sites. As we assumed that the BY-specific genetic basis of body size evolution would be global across multiple tissues, the co-upregulated BY DEGs (observed in at least two tissues) plus the BY PSGs were treated as a specific gene dataset. By screening the known functions of all these genes of interest, we obtained the final body size-related candidate genes. We also sampled some of the candidate genes via qRT-PCR to validate RNA-Seq transcriptome profiles in this study.

## 5. Conclusions

This study utilized RNA-Seq, along with a multiple comparisons, to identify DEGs, FEGs, and PSGs in three catfish species that are potentially associated with body size evolution. These three catfish species (BY, GM, and OS) belong to the same Asian Sisoridae family but have significantly divergent body sizes. As only wild populations and a few samples were available for the three species, we endeavored to minimize the possible age-induced and season-induced transcriptome variations by employing a preliminary age estimation for guidance and a unified strategy for sampling. However, other factors such as without ideally identical development stages, unified sex, and standard collection circumstances would also affect the exact expression profiles of these wild species, but this was beyond control.

**Figure 7:**
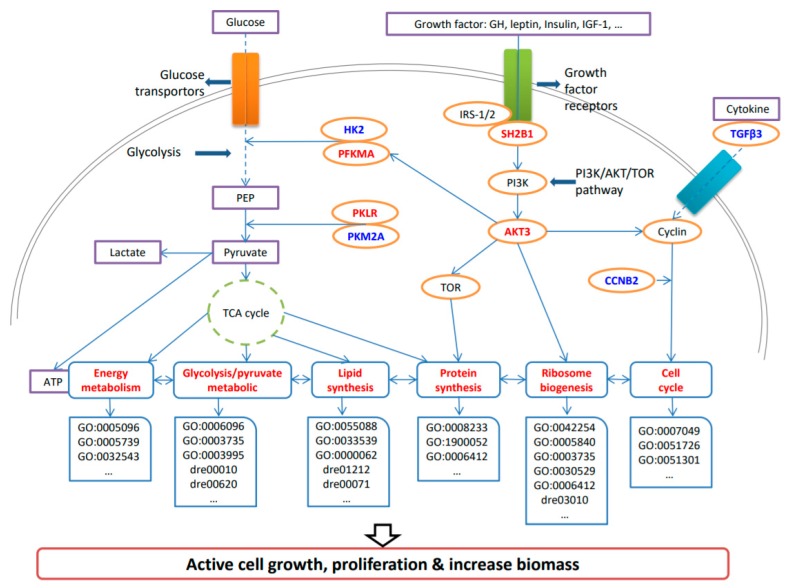
Schematic illustration of the main genetic clues revealed in this study that may be related to large BY body size when compared to GM and OS. Upregulated BY DEGs are shown in red (*SH2B1*, *AKT3*, *PFKMA*, and *PKLR*) and positively selected genes (PSGs) obtained from evolutionary analysis are shown in blue (*PKM2A, HK2, TGFβ3*, and *CCNB2*). The biological processes are denoted by red boxes (such as Ribosome biogenesis), with groups including many enriched GO terms or Kyoto Encyclopedia of Genes and Genomes (KEGG) pathways that are partially illustrated below the box. All of these clues could contribute to BY cell growth, proliferation, and increased biomass, thereby enabling the evolution of a much larger body size.

For these three species, we provided not only three high-quality transcriptomes but also found some BY-specific enriched pathways and functional clusters, and we screened 20 valuable candidate genes implicated in the significantly enlarged body size of BY. Among these candidate genes, two conserved and growth-related genes, *AKT3* and *SH2B1,* were found to be consistently upregulated in BY relative to GM and OS in all three tissue types. The effect of these two key growth modulators, *SH2B1* to *AKT3*, can promote cellular growth, proliferation, and biosynthetic processes at multiple levels. Furthermore, *AKT* exhibits downstream crosstalk that can promote ribosome biogenesis and protein synthesis (e.g., the *RPs* were detected to be enriched overall); activate the cell cycle to increase cell numbers (e.g., *TGFβ3* and *CCNB2* were under positive selection); and improve the pyruvate metabolic process (e.g., via upregulation of *PKLR* and positive selection of *PKM2A*), and thus modulate other downstream components associated with energy metabolic processes through the TCA cycle. These mechanisms may be critical to modulate cell growth and proliferation in BY and then promote its biomass increase (summarized in Figure 7). Because conceptually defined individual pathways are usually not functionally isolated but rather form whole networks by crosstalk actions, the above and other candidate genes identified in this study should be further examined. Furthermore, we also proposed an open hypothesis that habitat preferences may be involved in the body size divergence of Sisoridae catfish, according to field observations and morphological comparisons. The enhancement of the glycolysis/pyruvate metabolic process in the large-sized species of *Bagarius* would be critical if the utilization of glucose is found to be a limiting factor in the future.

## Figures and Tables

**Table 1:** Candidate genes (both differentially expressed genes (DEGs) and positively selected genes (PSGs)) highlighted in this study and their relative expressions in *Bagarius yarrelli* (BY) vs. *Glyptothorax macromaculatus* (GM), and BY vs. *Oreoglanis setiger* (OS).

No	Gene Name ^†^	Main Functional Keywords	BY vs. GM			BY vs. OS		
			Brain	Liver	Muscle	Brain	Liver	Muscle
1	*AKT3*	multi-functional protein kinases	2.71	2.46	3.6	2.07 ^§^	4.31	3.33
2	*SH2B1*	multi-functional adapter protein	10.97	2.38	2.74	10.47	2.72	10.82
3	*RPS3*	multi-functional ribosomal protein	18.09	16.3	8.01	17.59	16.07	16.84
4	*HSP70.2*	heat shock protein in wide processes	1.63	2.14	4.65	0.91 ^§^	2.41	2.55
5	*HIKESHI*	HSP70 carrier for heat-shock stress	2.69	0.97 ^§^	2.67	2.64	2.02	2.16
6	*HNRNPD*	telomere elongation	6.64	4.94	5.07	11.02	7.39	5.92
7	*RINT1*	telomere length control	5.92	2.19	2.9	3.64	2.29	3.82
8	*NOX5*	cell growth and apoptosis	6.69	5.85	6.44 ^§^	5.86	3.26	0.96 ^§^
9	*IMPDH2*	regulation of cell growth	6.32	1.39 ^§^	3.27	5.19	3.43	2.58
10	*PCGF1*	cycle progression and cell proliferation	2.76	2.84 ^§^	3.34	7.21	1.21 ^§^	5.05
11	*NRF1*	cellular growth and development	1.04 ^§^	1.46	1.99	3.25	3.43	3.5
12	*TRAF4*	cell survival and apoptosis	3.07	11.79	7.95	5.54	4.1	4.44
13	*USP9X*	neuronal cell migration and growth	3.59	3.44	3.12	1.5	1.14	0.88 ^§^
14	*TFB2M*	mitochondrial transcription factor	5.47	4.98	5	3.14	2.59	3.58
15	*TFDP2*	cell-cycle progression	9.08	2.01	1.50 ^§^	3.17	1.78	2.21 ^§^
16	*ACHE*	growth factor like actions	2.57	0.44 ^§^	0.40 ^§^	3.80	0.98 ^§^	1.63 ^§^
17	*PKM2A* ^‡^	glycolytic rate-limiting pyruvate kinase	2.49	0.21	1.81 ^§^	−0.65 ^§^	-0.82 ^§^	−0.38 ^§^
18	*TGF* *β3* ^‡^	cell growth and differentiation	0.29 ^§^	−0.11 ^§^	−0.22 ^§^	1.43 ^§^	-0.07 ^§^	0.01 ^§^
19	*CCNB2* ^‡^	regulation of cell cycle	−1.57 ^§^	−0.10 ^§^	−4.00	−2.64 ^§^	1.28 ^§^	−2.66 ^§^
20	*HK2* ^‡^	hexose metabolism	−3.57	−2.89	−4.01	−0.98 ^§^	−1.44 ^§^	−0.17 ^§^

Note: ^†^ Gene name abbreviations: *AKT3*: serine/threonine-protein kinases 3; *SH2B1*: adaptor protein 1; *RPS3*: ribosomal protein S3; *HSP70.2*: heat shock cognate 70-kd protein, tandem duplicate 2(hsp70.2); *HIKESHI*: HSP70 carrier protein Hikeshi; *HNRNPD*: heterogeneous nuclear ribonucleoprotein D; *RINT1*: RAD50 interactor 1; *NOX5*: NADPH oxidase, EF-hand calcium binding domain 5; *IMPDH2*: IMP (inosine 5’-monophosphate) dehydrogenase 2; *PCGF1*: polycomb group ring finger 1; *NRF1*: nuclear respiratory factor 1; *TRAF4*: TNF receptor-associated factor 4; *USP9X*: probable ubiquitin carboxyl-terminal hydrolase FAF-X; *TFB2M*: transcription factor B2, mitochondrial; *TFDP2*: transcription factor Dp-2; *ACHE*: acetylcholinesterase; *PKM2A*: pyruvate kinase, PKM; *TGFβ3*: transforming growth factor beta-3; *CCNB2*: G2/mitotic-specific cyclin-B2; *HK2*: Hexokinase-2. ^‡^ indicate PSGs, while DEGs are unmarked; the values are shown as Log_2_ FC (Fold Change) with false discovery rate (FDR) < 0.05 or ^§^ FDR > 0.05.

## Supplementary Materials

Supplementary materials can be found at https://www.mdpi.com/1422-0067/20/4/944/s1.
