# Supplementary material for: Insights into Body Size Evolution: A Comparative Transcriptome Study on Three Species of Asian Sisoridae Catfish
Source: Int J Mol Sci. 2019 Feb 21;20(4):944. doi: 10.3390/ijms20040944 (PMC6412271; doi:10.3390/ijms20040944)
Supplement: Supplementary file 1 [file ijms-20-00944-s001.zip › ijms-443256-supplementary/Figure S1-S2.pdf]

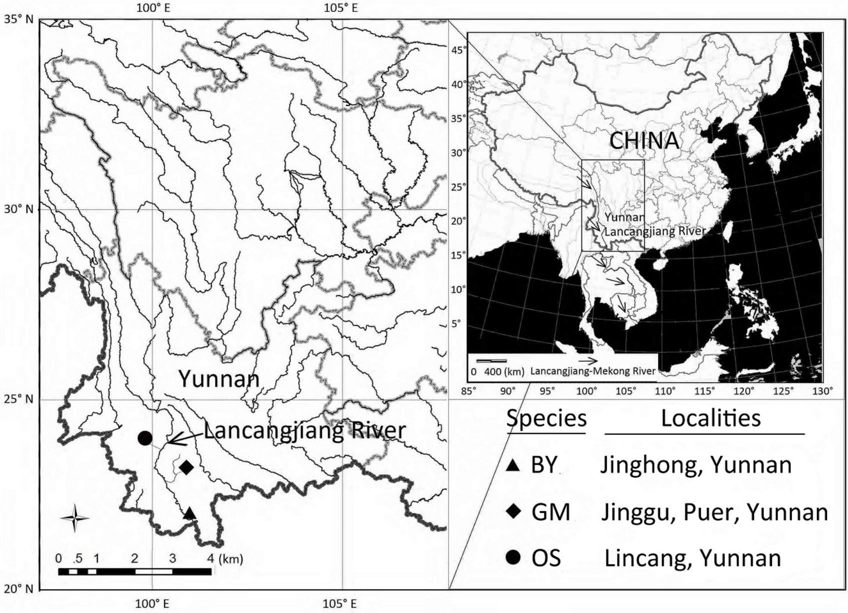

Figure S1. The collection localities of BY, GM and OS used in this study.

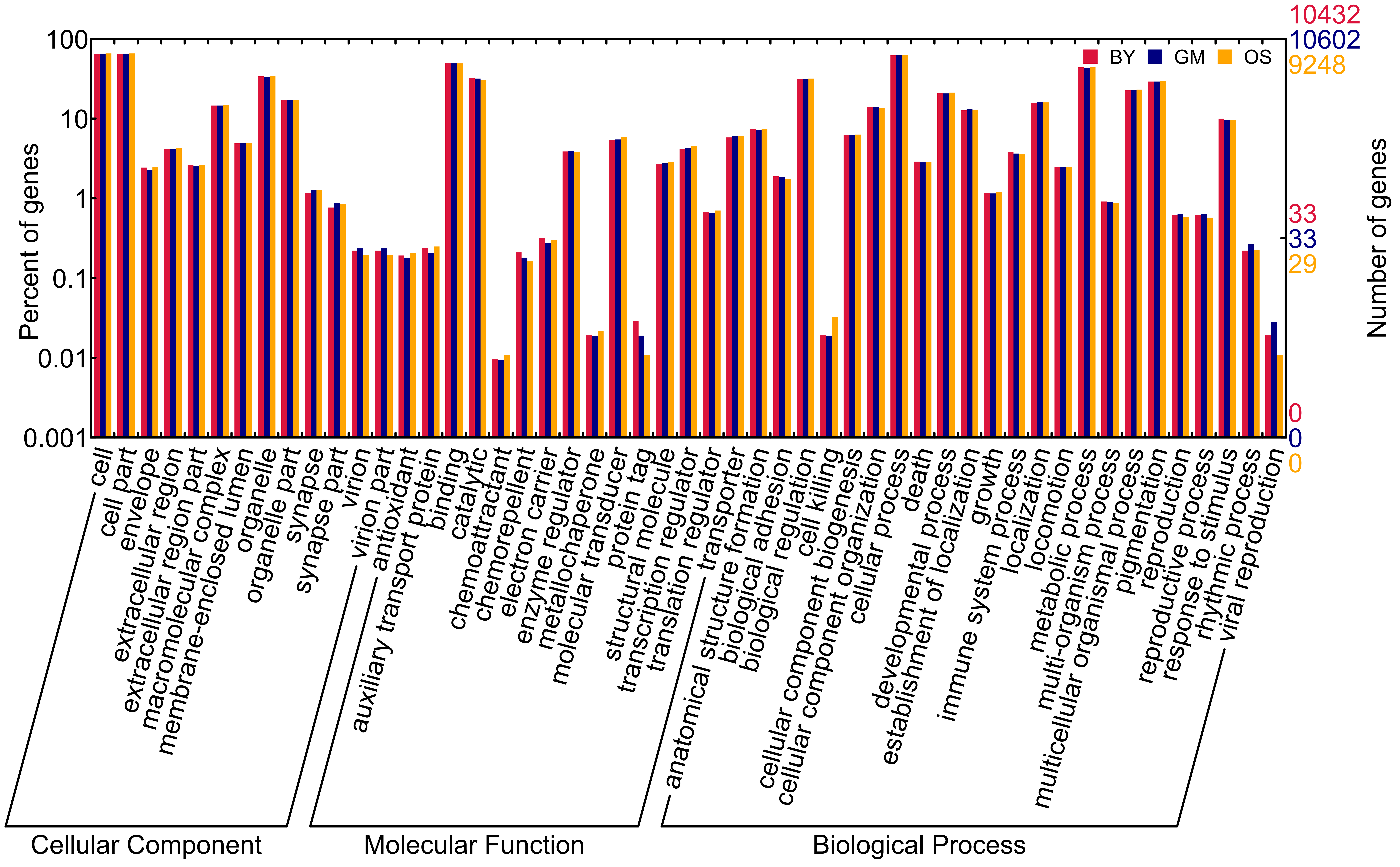

Figure S2. GO classification of annotated genes in the BY, GM, and OS groups.
